# Alterations in synaptonemal complex coding genes and human infertility

**DOI:** 10.7150/ijbs.67843

**Published:** 2022-02-21

**Authors:** Fengguo Zhang, Mengfei Liu, Jinmin Gao

**Affiliations:** Institute of Biomedical Sciences, College of Life Sciences, Key Laboratory of Animal Resistance Biology of Shandong Province, Shandong Normal University, Jinan, Shandong, 250014, China.

**Keywords:** Meiosis, synaptonemal complex, premature ovarian insufficiency, non-obstructive azoospermia, infertility

## Abstract

About 10% of reproductive-aged couples suffer from infertility. However, the genetic causes of human infertility cases are largely unknown. Meiosis produces haploid gametes for fertilization and errors in meiosis are associated with human infertility in both males and females. Successful meiosis relies on the assembly of the synaptonemal complex (SC) between paired homologous chromosomes during the meiotic prophase. The SC is ultrastructurally and functionally conserved, promoting inter-homologous recombination and crossover formation, thus critical for accurate meiotic chromosome segregation. With whole-genome/exome sequencing and mouse models, a list of mutations in SC coding genes has been linked to human infertility. Here we summarize those findings. We also analyzed SC gene variants present in the general population and presented complex interaction networks associated with SC components. Whether a combination of genetic variations and environmental factors causes human infertility demands further investigations.

## Introduction

It is estimated that about 8% to 12% of reproductive-aged couples worldwide suffer from infertility 1, 2. Among these affected couples, the incidence of disorders seems to distribute equally between males and females 3, 4. Azoospermia is a major cause of male infertility, affecting about 10-15% of infertile men 5-7. Azoospermia contains two subcategories, obstructive azoospermia (OA) and non-obstructive azoospermia (NOA). Patients with the former may have normal spermatogenesis in the testis, but the excurrent ducts between the rete testis and the ejaculatory ducts are obstructed, causing the absence of sperm in the semen. In patients with NOA, testicular spermatogenesis is abnormal, and sperm cannot be produced 8. Ovulation disorders account for most cases of female infertility. Premature ovarian failure (POF), also referred to as premature ovarian insufficiency (POI), is characterized by absent menarche (primary amenorrhea) or premature depletion of ovarian follicles before the age of 40 (secondary amenorrhea) 9. Although the causes of infertility can be highly heterogeneous, genetic factors likely contribute a lot. Indeed, studies of infertile pedigrees and cohorts revealed the genetic causes of infertility in males 10-12 and females 9, 13.

Meiotic cell division is required for all sexually reproductive organisms to generate haploid gametes, i.e., sperm and eggs. Defects or failure in meiosis can cause NOA or POI in humans 14, 15. To produce haploid gametes, DNA is replicated only once, and the cell undergoes two consecutive divisions, meiosis I (separation of homologous chromosomes) and meiosis II (separation of sister chromatids) 16. To set the ordered meiotic chromosome segregation process, a series of critical meiosis-specific events occur during meiotic prophase, including the formation of double-strand break (DSB), homologous pairing, synapsis, inter-homologous recombination, and crossover formation. Chromosomes undergo dramatic dynamic changes during the prophase. According to their morphology, the meiotic prophase can be divided into five substages: leptotene, zygotene, pachytene, diplotene, and diakinesis. During the early meiotic prophase (leptotene and zygotene), homologs pair and synapse. A proteinaceous structure called the synaptonemal complex (SC) starts to assemble between the paired homologous chromosomes. The completion of SC formation along the entire lengths of the homolog pairs allows the cells to progress into pachytene. During diplotene, chromosomes desynapse, and the SC starts to disassemble 17. For more comprehensive information, we refer the interested readers to recent reviews focusing on various aspects of meiosis, including the organization and regulation of the SC 18-20, meiotic recombination and crossover control 21-23.

Studies from various organisms have revealed the critical roles of the SC in meiotic progression. A list of SC gene mutations has been identified in infertile patients, which show NOA or POF/POI phenotypes. In this review, we focus on the role of the SC in meiosis and summarize how mutations in SC coding genes are linked to human infertility.

## The tripartite structure and self-assembly properties of the SC

**Figure 1:**
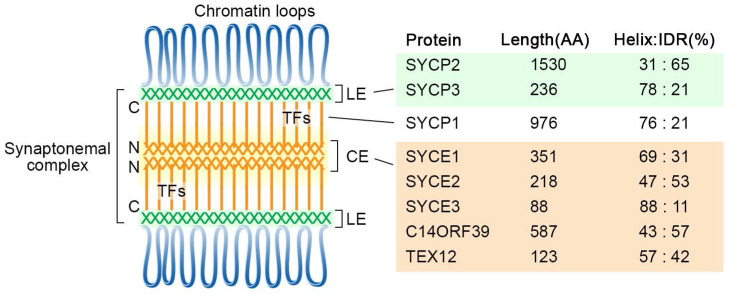
** Diagram of the tripartite SC structure and its components in humans**. SC proteins are mainly composed of α-helixes and intrinsically disordered regions, and their percentages are indicated for each protein. The locations of the N- and C-termini of SYCP1 are labeled. CE, central elements; LE, lateral elements; TFs, transverse filaments; IDR, intrinsically disordered region.

The chromosomes are organized as loop-axis structures during the meiotic prophase. The formation of chromosome axis provides the framework for SC assembly, and the axis is then referred to as lateral elements (LEs). The fully assembled SC consists of two parallel LEs, a central element (CE) in the middle and the bridging transverse filaments (TFs) (Figure 1). The CE and TFs are together called the central region. In mammals, eight meiosis-specific proteins have been identified as the SC components, including two LE components, SYCP2 and SYCP3, five CE proteins SYCE1, SYCE2, SYCE3, TEX12, and C14ORF39/SIX6OS1, and one TF component SYCP1 17.

The formation of the SC lateral elements likely requires the self-assembly property of SYCP proteins in mammals. Structural analyses showed that human SYCP3 forms tetramers, which can further assemble into filamentous structures and have DNA binding properties 24, 25. Co-purification and biochemical analysis suggest SYCP3 and the C-terminal coiled-coil region of SYCP-2 form hetero-tetramers, which can oligomerize to form the LEs core 26.

Human SC central region proteins also exhibit self-assembly properties. TF protein SYCP1 serves as the core architecture of the SC. X-ray crystallographic and biophysical studies showed that SYCP1 has an obligate tetrameric structure, and through two self-assembly sites, assembles into a zipper-like lattice, in which the N-terminal sites assemble in the midline, and the C-terminal tails face towards the chromosome axis 27. Central element proteins SYCE2 and TEX12 can form 2:2 and 4:4 building blocks, which further assemble into 2-nm and 4-nm fibers or bundled fibers through end-to-end association 28. Multivalent interactions apparently mediate the assembly of the SC central elements. SYCE1 can self-dimerize 29, and this self-dimerization property can be altered to form 1:1 heterodimer with C14ORF39/SIX6OS1 30. Mutations disrupting SYCE1-C14ORF39 interactions have been linked to infertility in humans and mice 30, 31. SYCE3 also exhibits a self-assembly property 32, and interaction with SYCE1 has also been revealed 33. However, it remains to be determined how SYCE1 can alter SYCE3 self-assembly property. Moreover, it will be essential to examine the SC self-assembly properties when all components are provided.

The highly ordered SC structure and the self-assembly properties of SC components have promoted the hypothesis that SC structure is static. However, studies from model organisms suggest that the SC is dynamic 18. Its components consistently come on and off from chromosomes during early meiotic prophase, and this dynamic property can be modulated by meiotic recombination and crossover (CO) designation 34, 35. Moreover, polycomplexes formed by SC central region proteins in axis defective mutants in the nematode *Caenorhabditis elegans* also exhibit dynamic properties 36. However, due to technical difficulties, the dynamic property of the SC has not been able to be directly examined in mammalian meiosis.

## The SC has conserved roles in meiotic recombination and CO regulation

The tripartite proteinaceous ultrastructure of the SC provides the spatial scaffold for homologous recombination and CO formation. Studies from multiple organisms showed that SC components play important roles in various steps of meiotic recombination, including recombination initiation, feedback control of DSB formation, choice of DSB repair pathways, and the control of CO number and distribution 18, 37. For example, meiotic chromosome axial components HTP-3 in *C. elegans*, and Sycp2 in zebrafish, are required for recombination initiation 38, 39. DSB repair proteins and pro crossover factors can be recruited to the SC in *C. elegans* 40. Homolog engagement and synapsis, in turn, inhibit further DSB formation by removing DSB machinery from the chromosomes in *C. elegans* and yeast 35, 41. The occurrence of a CO at one locus is accompanied by a decreased probability that a second CO will occur nearby, a conserved phenomenon that is referred to as CO interference 42. Interestingly, the meiotic chromosomal axis length is inversely correlated to CO rates across species 43-45. Proteins that regulate meiotic chromosome axis length also regulate CO frequencies 46, 47. Although the chromosome axis was considered the major compartment controlling recombination event number and distribution 21, 37, recent findings suggest that the SC central region also plays an essential role. For example, in budding yeast, SC central region components are involved in modulating CO pathways and feedback control of DSB formation 41, 48. In *C. elegans*, SC central region promotes the recruitment of pro-CO factors, and the intact SC is required for wild-type level of CO interference 40, 49. Moreover, in *Arabidopsis*, SC TFs are required to regulate CO rates and probably interference 50, 51. These observations established the conserved functions of the SC in controlling CO formation. However, it is still not fully understood how each SC component contributes to such CO control in humans.

## Unconventional functions of SC components

In addition to controlling homologous recombination and CO formation during early meiotic prophase, SC proteins may also have additional roles during meiosis. Studies from various organisms suggest that SC components do not dissociate from the chromosomes simultaneously upon pachytene exit. Instead, some components are retained until late meiotic prophase or metaphase I 18, 52. Studies in worms suggest that the maintenance of the SC on chromosome subdomains during late prophase is required for chromosome remodeling 53, and HORMA domain proteins are required for proper centriole engagement during spermatocyte meiosis 54. The transverse filament protein Zip1 in yeast was found promoting centromere pairing 55. In mouse spermatocytes, SC components also promote centromere pairing. A tripartite SC structure was observed at the centromere persisting until diplotene, and LE protein SYCP3 remains associated with the sister centromeres until anaphase I or telophase I 56-58. The centromere retention of SYCP3 was also suggested to promote monopolar orientation of sister kinetochores during meiosis I 58. Moreover, shugoshin can promote centromere pairing and segregation of nonexchange homolog chromosomes, potentially by protecting the centromeric SC 59. These observations suggest SC components may have multiple functions during meiosis.

## Synapsis defects and infertility

While synapsis and meiotic recombination are essential for establishing chiasmata and preventing aneuploidy, synapsis defects during early meiotic prophase can cause meiotic arrest and apoptosis, which are more directly associated with infertility in mammals. Meiotic sex chromosome inactivation (MSCI) is an essential process in mammalian male germline development, and the failure to initiate MSCI causes the complete arrest and elimination of germ cells during the mid pachytene stage 60, 61. Consistently, epigenetic dysregulation was observed in mammalian male meiosis with synapsis and recombination defects 62. Mechanistically, meiotic arrest caused by SC mutations can be attributed to the failure to repair programmed DSBs, defects in MSCI, and/or meiotic silencing of unsynapsed chromosomes 63, 64.

So far, mutant mouse models are available for all known SC components, and their phenotypes are summarized in Table 1. Null mutants for SC central region components show infertility in both males and females. However, null mutations of LEs (SYCP2 and SYCP3) cause different phenotypes between the genders. While the males are infertile, females are subfertile and have a reduced number of offspring 65, 66. Although the detailed mechanism underlying such difference requires further investigation, it may be related to different stringencies of checkpoint activation in male and female meiosis.

## Infertility-related SC gene mutations in humans

Given the conserved roles of the SC in meiosis and infertility phenotypes observed for mice carrying SC mutations, it is expected that mutations in SC coding genes are linked to human infertility. Indeed, with whole-genome/exome sequencing and the creation of mouse models, a list of SC coding gene mutations has been identified and linked to human infertility. So far, infertility-related mutations have been identified in four SC coding genes, including *SYCP2*, *SYCP3*, *SYCE1*, and *C14ORF39* (Table 2).

SYCP2 and SYCP3 are the core axis proteins in mammals and have a direct interaction 26, 67. *SYCP2* has 47 exons and encodes a 1350 amino acid protein product, while *SYCP3* has 8 exons and encodes a 236 amino acid protein product. Several studies revealed that mutations in their coding genes are associated with human infertility in both males and females. Through exome sequencing (ES) in a cohort of 627 male patients with diverse infertility phenotypes, three heterozygous *SYCP2* frameshift mutations were identified in three azoospermia patients. Such frequency is significantly higher than that in the general population, suggesting the tight association between *SYCP2* mutations and male infertility 68, 69. *SYCP3* also shows a high mutation frequency in infertile patients. In a mixed ethnic origin of 23 couples with recurrent miscarriages, a heterozygous change c.548T>C was detected in *SYCP3*, which caused a missense mutation in the coiled-coil domain of SYCP3 70. In a separate study, two out of 26 women with recurrent pregnancy loss of unknown cause were found to carry independent heterozygous mutations in *SYCP3*, i.e., c.553-16_19del and c.657T>C, both of which disrupt normal splicing and possibly result in C-terminally truncated proteins 71. By analyzing a small group of 19 azoospermic patients with maturation arrest, a 1bp deletion (643delA) in *SYCP3* that results in a premature stop codon and truncation of its C-terminal coiled-coil region was identified in two patients 72. These identified mutations mainly disrupt the C-terminus of SYCP3, and their co-expression with their wild-type counterpart interfered with SYCP3 fiber formation in cultured cells 71, 72.

Mutations in two SC central element genes have also been linked to human infertility, i.e., *C14ORF39* and *SYCE1*. Interestingly, protein interaction analysis suggests their protein products form heterodimers 31, which may be critical for SC functions *in vivo*. So far, six mutations in *C14ORF39* were identified in infertile males and females by whole-exome sequencing 73-75. These mutations include a frameshift mutation (c.204_205del) from an NOA/POI family, a nonsense mutation (c.958G>T) and a splicing mutation (c.1180-3C>G) from two unrelated individuals with meiotic arrest 73, a nonsense mutation (c.508C>T) from a sporadic POI patient and a frameshift mutation (c.135_136del) from a sporadic NOA patient 75, and a frameshift mutation (c.207_210delTGAG) from POI patients 74. These mutations were predicted to destroy C14ORF39-SYCE1 polycomplex formation 73 or significantly accelerate protein degradation 75.

A growing list of pathogenic mutations has also been identified in *SYCE1*. Two splicing mutations of *SYCE1* were identified in two independent autosomal recessive NOA families 30, 76, 77. SYCE1 protein could not be detected in patients carrying c.197-2 A > G mutation in the testicular tissue. Another mutation c.375-2A>G was predicted to result in the deletion of SYCE1 126-155 amino acid region, which locates in its coiled-coil core. Moreover, a nonsense *SYCE1* mutation c.613C>T was identified in a consanguineous Israeli Arab family with POI 78. This nonsense mutation and the splicing mutation c.375-2A>G were predicted to interrupt the binding interfaces between SYCE1 and C14ORF39/SIX6OS1 30, highlighting the importance of their interactions. Indeed, mice carrying the analogous mutations exhibit synapsis defects and are infertile for both females and males 79. In addition to these mutations, recent studies have identified a missense mutation (c.475G>A), two frameshift mutations (c.689_690del and c.658_662delGAGGG) in POI patients 74, 75, and a splicing mutation (c.271+2T>C) in sporadic NOA patients 75.

In addition to mutations within SC genes, copy number variants and translocation-mediated gene dysregulation may also be linked to human infertility. For example, multiple groups have revealed the associations of POI with deletions or duplications of chromosome regions encompassing SYCE1 80-85. Moreover, a homozygous deletion of the whole SYCE1 gene was detected in a targeted next-generation sequencing study of 668 Chinese NOA patients 86. While the pathogenicity remains to be determined, duplication of SYCE1 has also been observed in POI patients 82. Furthermore, translocation-mediated SYCP2 overexpression has been suggested to cause male infertility 68.

Interestingly, although mutations in both LE and CE coding genes can cause infertility, most identified mutations in LE genes have a dominant-negative effect, while mutations in CE genes are generally homozygous recessive mutations 87. Such differences may be related to haploid insufficiency and/or the incorporation of mutated proteins into the SC. The mutated form of LE proteins, which are mostly C-terminally truncated, may still be incorporated into the meiotic chromosome axis, interfering with normal synapsis. In another scenario, a single copy of wild-type LE gene expression may not provide enough protein product required for normal synapsis and meiotic progression, exhibiting haploid insufficiency. Regarding CE proteins, mutations may directly disrupt its binding with other SC proteins, or SC protein complexes/subunits containing the mutated forms fail to be incorporated into the SC in heterozygous mutants, thus having little impact on normal synapsis. Meiosis may be more tolerant to changes in central region protein levels than to changes in LE protein levels. So far, infertility-related SC gene mutations are limited to four SC genes (*SYCP2*, *SYCP3*, *C14ORF39*, and *SYCE1*). However, given the critical roles of the other SC components in meiotic progression, it would not be surprising to identify infertility-related mutations in the other SC genes as the number of studied cases increases. Moreover, although most infertility-related SC mutations were reported only for a specific gender, mutations within the same SC gene were usually found in both male and female infertile patients. Those reported infertility-related SC mutations may cause infertility in both genders.

## Frequencies of SC gene mutations in the general population

SC genes only express in the germline during meiosis, and they are unlikely to be required for normal human development 88. Moreover, some SC gene mutations might be recessive, and they do not cause infertility when they are carried in heterozygotes. These facts imply that the general population may carry a certain number of infertility-related SC mutations. To assess this, we examined the allele frequencies of SC gene variants in the Genome aggregation database (gnomAD, https://gnomad.broadinstitute.org), which includes WES data of 123,136 individuals and WGS data of 15,496 individuals of different ethnic origins 89, and may serve as a genome database for the general population regarding fertility.

**Figure 2:**
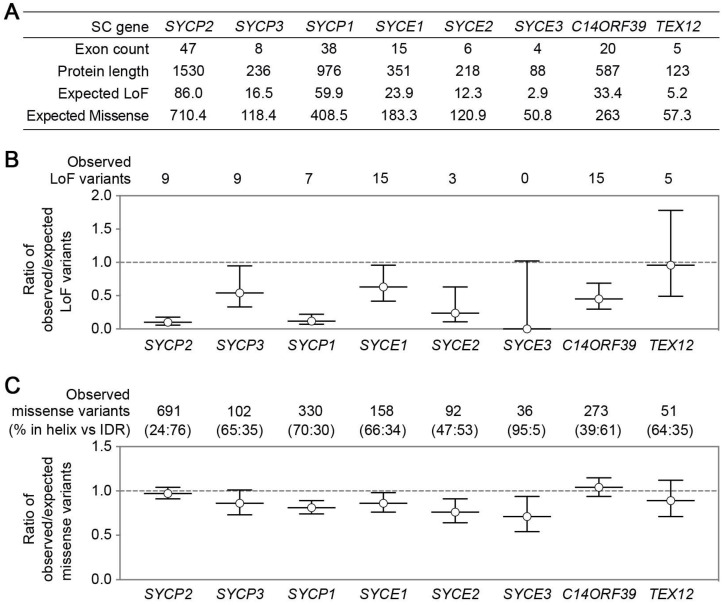
** SC gene variants in the general population.** SC gene variants data was collected from the gnomAD database 69. **(A)** A table summarizes SC gene exon counts, protein product lengths, and the expected number of loss-of-function (LoF) or missense variants. **(B)** The ratio of the observed/expected number of LoF variants in SC genes. Error bars represent a 90% confidence interval. The numbers of observed LoF variants are indicated on the top. **(C)** The ratio of the observed/expected number of missense variants in SC genes. Error bars represent a 90% confidence interval. The numbers of observed missense variants and their distribution in α-helix or IDR regions (shown as percentages in parentheses) are indicated on the top. The expected variant numbers and ratio of observed/expected variants with confidence intervals were determined with a depth corrected mutation probability reported in 89.

SC genes have significant differences in exon counts and protein product sizes, resulting in the differences in their expected numbers of loss-of-function (LoF) or missense variants (Figure 2A), which were determined with a depth corrected mutation probability 89. Four genes with the most observed LoF variants are *SYCE1*, *C14ORF39*, *SYCP2*, and *SYCP3*, corresponding to the four reported genes with infertility-related mutations. The ratio of observed/expected (OE) number of variants measures how tolerant a gene is to a certain class of variation, and low OE values indicate strong intolerance. Surprisingly, SC genes likely have distinct LoF intolerance levels (Figure 2B). LE coding gene *SYCP2* and TF coding gene *SYCP1* have significantly lower OE values than the other SC genes, suggesting that they may be more intolerant to LoF than other SC genes. It will be interesting to understand why different SC genes have different LoF intolerance. The low tolerance to LoF of SYCP2 and SYCP1 may have several causes. One explanation is that some of their functional domains are located near the C-terminal, making most truncated proteins unfunctional. Additionally, the function of these proteins in SC formation cannot be easily compensated by other SC components. Moreover, they may not only participate in SC formation but also have other functions (e.g., centromere paring).

Compared to LoF, missense OE values show less variation among the SC genes (Figure 2C). *SYCP2* and *C14ORF39* have slightly higher OE values than the other SC genes. Interestingly, proteins encoded by these two genes contain the longest intrinsically disordered regions (IDRs), and the majority of the missense variants locate in their IDRs. SC IDRs are likely more tolerant than helix regions to missense mutations. This is consistent with the observation that helix regions of the SC proteins usually mediate stable protein-protein interactions and are thus critical for SC functions. However, recent findings have also highlighted the important roles of IDRs in SC regulation in *C. elegans* 53, 90. Further studies are required to identify elements in IDRs that are critical for SC regulations in various organisms, including in humans.

## SC protein interaction networks

**Figure 3:**
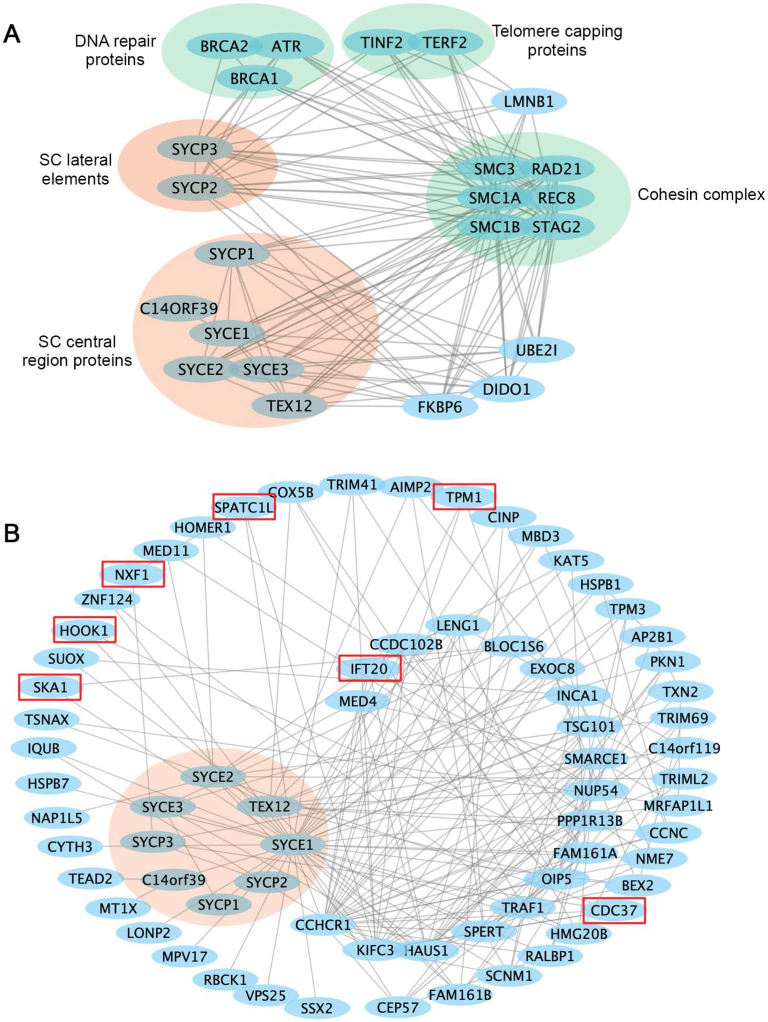
** Interaction networks of the SC proteins. (A)** Functional protein association networks of the SC proteins were obtained from the STRING database (https://string-db.org). Presented are known interactions, which were sourced from curated databases or were experimentally determined. **(B)** SC protein interaction networks mapped in the human reference interactome database (www.interactome-atlas.org). Proteins not expressed in the testis or ovary were removed. Proteins confirmed or predicted to be required for meiosis or gametogenesis are indicated by red boxes. Interaction networks were visualized by Cytoscape.

Cellular activities are mediated by complex interactome networks of physical, biochemical, and functional interactions of the proteins 91. Complex biological interactions and cellular networks underlie most genotype to phenotype relationships 92. More and more studies have revealed physical and functional associations between the SC and other nuclear proteins. STRING database presents known and predicted protein-protein interactions, which include direct physical and indirect functional associations. Analysis of the SC protein interaction networks in STRING revealed their connections with cohesin complexes, proteins involved in DNA repair, telomere capping, ubiquitination, etc. (Figure 3A). Combined gene variants within this interaction network may cause severe impacts on human fertility.

The study in humans and model organisms has provided insights into how internal interactions between SC components mediate SC formation. However, it remains to be explored how SC assembly and functions are regulated by non-structural proteins, and what biological processes are regulated by SC components. Available protein interaction databases may provide clues about how SC proteins interact with the rest of the proteome. To this end, we extracted an SC binary interaction network from the human reference interactome, which covers about 90% of the protein-coding genome 93 (Figure 3B). Interactome analysis revealed that SC proteins vary a lot regarding the number of binding partners, with the central element proteins SYCE1 having the most binding partners. Interestingly, LoF mutations in SYCE1 are most frequent in the general population, and the associations between SYCE1 mutations and infertility are also well established. Thus, it will be essential to evaluate how disruption of SYCE1 interactome may contribute to human infertility.

Within the SC interactome, several proteins are potentially involved in meiotic cell division or gametogenesis, including the spindle and kinetochore-associated protein 1(SKA1) 94, nuclear RNA export factor 1 (NXF1) 95, cell division cycle 37 (CDC37) 96, the coiled-coil alpha-helical rod protein 1 (CCHCR1) 97, intraflagellar transport protein 20 (IFT20) 98, spermatogenesis and centriole-associated 1 like (SPATC1L) 99, and hook microtubule tethering protein 1 (HOOK1) 100. It will be important to confirm whether these interactions exist *in vivo* and whether abnormal interactions between them may contribute to human infertility.

It is well established that SYCP2 and SYCP3 form the LE core, and the C-terminus of SYCP1 faces towards LEs. Thus, the C-terminus of SYCP1 was expected to interact with LEs. However, direct interactions between SYCP1 and LEs have not been able to be detected. This raises the possibility that SC central region proteins may interact with the LEs through multivalent weak interactions, which may not be detected by conventional methods. Remarkably, SC central region proteins are evolutionarily divergent. The application of multivalent weak interactions to mediate synapsis may underlie the rapid evolution of SC components 101.

## Future perspectives

Infertility remains a severe problem affecting couples wishing to have children. It is ranked third in major diseases after tumors and cardiovascular diseases. The cause of infertility is very complicated. For about one-fifth of the cases, the causes are still unknown. Genome or exome sequencing for infertile patients combined with mouse models will still be a powerful strategy to identify the genetic causes of at least some types of infertility. The SC plays an essential role during meiosis to produce the haploid gametes for fertilization. Understanding how alterations in SC genes are linked to infertility will improve the diagnosis, prevention, and treatment of this disease. Addressing the following outstanding questions will help us to make a better connection between the SC and human fertility.

Have all human SC components been identified? Functional redundancy may hinder the discovery of new SC components through phenotype analysis. Given the conservation of SC components in mammals, proteomic analysis in mice or primates will help to identify new SC components or its binding proteins.

How is the SC assembled into the functional tripartite structure? Although structural and biochemical studies have provided insights into the self-assembly properties of SC subcomplexes, how the entire SC is assembled remains not fully understood. For example, how are the SC central region proteins organized when all components are present? How do the SC central region proteins interact with the lateral elements? What are the roles of the IDRs in SC assembly? Characterizing the precise interaction network in the SC will help identify the critical sequence elements essential for SC formation.

Does the human SC have dynamic properties? While the dynamic properties of the SC have been observed in multiple organisms, whether the SC in humans has the same properties remain to be determined. Addressing this will help to understand how the SC performs its conserved functions in crossover regulation.

Can combined gene variants produce synergistic effects? Attention should be paid when multiple genetic variants are observed in physically or functionally associated components, although the single variant might not look harmful. Importantly, with a growing set of individual genomes available, humans are likely emerging as a direct biological model to study rare genetic variations and combinations, which are actually more difficult to explore in other systems.

Can SC gene variants combine with internal or external factors to cause infertility? Some SC gene mutations have been shown to cause severe meiotic defects only when grown at suboptimal temperatures in worms 90. Human infertility might be caused by a combination of SC gene variants with internal or external factors, such as diseases, aging, chemical exposure, and extreme temperatures. Clarifying their associations with human infertility will be of great significance to reproductive health.

## Figures and Tables

**Table 1:** Phenotypes of SC mutant mouse models

SC Gene	Mutation type	Gender	Gonad size	Fertility	Reported meiotic defects	Reference
*SYCP2*	Partial deletion (exons 39-43)	Male	Small	Infertile	Synapsis failure, abnormal AEs, increased apoptosis	66
		Female	NA	Subfertile	Synapsis failure	66
*SYCP3*	*Null*	Male	Small	Infertile	Synapsis failure, zygotene arrest, increased apoptosis	102
		Female	NA	Subfertile	Failure in chiasma formation, aneuploidy	65
*SYCP1*	*Null*	Male	Small	Infertile	Synapsis failure, unrepaired DSBs, failure in CO formation, increased apoptosis	103
		Female	Small	Infertile	Increased apoptosis	103
*SYCE1*	*Null*	Male	Small	Infertile	Synapsis failure, unrepaired DSBs, increased apoptosis	88
		Female	Small	Infertile	Synapsis failure, unrepaired DSBs, increased apoptosis	88
*SYCE2*	*Null*	Male	Small	Infertile	Synapsis failure, unrepaired DSBs, increased apoptosis	104
		Female	Small	Infertile	Synapsis failure,unrepaired DSBs, increased apoptosis	104
*SYCE3*	*Null*	Male	Small	Infertile	Synapsis failure, unrepaired DSBs, increased apoptosis	105
		Female	Small	Infertile	Synapsis failure, unrepaired DSBs, increased apoptosis	105
*SIX6OS1*	*Null*	Male	Small	Infertile	Synapsis failure, unrepaired DSBs, increased apoptosis	31
		Female	No follicles	Infertile	NA	31
*TEX12*	*Null*	Male	NA	Infertile	Partial synapsis, unrepaired DSBs, failure in CO formation	106
		Female	Small	Infertile	Partial synapsis, unrepaired DSBs, failure in CO formation, increased apoptosis	106

Abbreviation: NA, not available; AE, axial element; CO, crossover.

**Table 2:** SC gene mutations identified in infertile patients

Gene	Location	DNA change	AA change	Heterozygosity	Origin	Disease/Phenotype	Reference
*SYCP2*	20q13.33	c.2022_2025del	p.Lys674AsnfsTer8	Heterozygous	-	cryptozoospermia	68
*SYCP2*	20q13.33	c.2793_2797del	p.Lys932SerfsTer3	Heterozygous	-	cryptozoospermia	68
*SYCP2*	20q13.33	c.3067_3071del	p.Lys1023LeufsTer2	Heterozygous	-	azoospermia	68
*SYCP3*	12q23.2	c.548T>C	p.Ile183Thr	Heterozygous	Caucasian/Arabic mixed	recurrent miscarriage	70
*SYCP3*	12q23.2	c.553-16_19del	-	Heterozygous	Japanese	RPL	71
*SYCP3*	12q23.2	c.643delA	-	Heterozygous	Hispanic/Arabs	azoospermia	72
*SYCP3*	12q23.2	c.657T>C	-	Heterozygous	Japanese	RPL	71, 107, 108
*SYCE1*	10q26.3	c.197-2A>G	Splicing site	Homozygous	Iranian	NOA	30, 76
*SYCE1*	10q26.3	c.271+2T>C	Splicing site	Homozygous	Han Chinese	NOA	75
*SYCE1*	10q26.3	c.375-2A > G	Splicing site	Homozygous	Iranian	NOA	30, 77
*SYCE1*	10q26.3	c.475G>A	p.Glu159Lys	Compound heterozygous	Han Chinese	POI	75
*SYCE1*	10q26.3	c.613C>T	p.Gln205*	Homozygous	Israeli	POF	30, 78, 79
*SYCE1*	10q26.3	c.658_662delGAGGG	p.Glu220ProfsTer5	Heterozygous	Mixed	POI	74
*SYCE1*	10q26.3	c.689_690del	p.Phe230Serfs*21	Compound heterozygous	Han Chinese	POI	75
*SYCE1*	10q26.3	gross deletion (>4000bp)	-	Homozygous	Chinese	POI	85
*SYCE1*	10q26.3	whole gene deletion	-	Homozygous	Chinese	NOA	86
*C14orf39*	14q23.1	c.135_136del	p.Lys45Asnfs*5	Homozygous	Han Chinese	NOA	75
*C14orf39*	14q23.1	c.204_205del	p.His68Glnfs*2	Homozygous	Pakistani	NOA/POI	73
*C14orf39*	14q23.1	c.207_210delTGAG	p.Ser69ArgfsTer53	Heterozygous	Mixed	POI	74
*C14orf39*	14q23.1	c.508C>T	p.Arg170*	Homozygous	Han Chinese	POI	75
*C14orf39*	14q23.1	c.958G>T	p.Glu320*	Homozygous	Chinese	meiotic arrest	73
*C14orf39*	14q23.1	c.1180-3C>G	Splicing site	Homozygous	Chinese	meiotic arrest	73

Abbreviation: RPL, recurrent pregnancy loss; NOA, non-obstructive azoospermia; POF, premature ovarian failure; POI, premature ovarian insufficiency.

## Abbreviations

SC: synaptonemal complex
OA: obstructive azoospermia
NOA: non-obstructive azoospermia
POF: premature ovarian failure
POI: premature ovarian insufficiency
LE: lateral element
CE: central element
TF: transverse filament
CO: crossover
LoF: loss-of-function
IDR: intrinsically disordered region

## References

[1] Vander Borght M, Wyns C (2018). Fertility and infertility: Definition and epidemiology. Clin Biochem.

[2] Pisarska MD, Chan JL, Lawrenson K, Gonzalez TL, Wang ET (2019). Genetics and Epigenetics of Infertility and Treatments on Outcomes. J Clin Endocrinol Metab.

[3] Datta J, Palmer MJ, Tanton C, Gibson LJ, Jones KG, Macdowall W (2016). Prevalence of infertility and help seeking among 15 000 women and men. Hum Reprod.

[4] Agarwal A, Mulgund A, Hamada A, Chyatte MR (2015). A unique view on male infertility around the globe. Reprod Biol Endocrinol.

[5] Tang D, Lv M, Gao Y, Cheng H, Li K, Xu C (2021). Novel variants in helicase for meiosis 1 lead to male infertility due to non-obstructive azoospermia. Reprod Biol Endocrinol.

[6] Liu M, Yang Y, Wang Y, Chen S, Shen Y (2021). The mutation c.346-1G > a in SOHLH1 impairs sperm production in the homozygous but not in the heterozygous condition. Hum Mol Genet.

[7] Wen L, Liu Q, Xu J, Liu X, Shi C, Yang Z (2020). Recent advances in mammalian reproductive biology. Sci China Life Sci.

[8] Li Z, Huang Y, Li H, Hu J, Liu X, Jiang T (2015). Excess of rare variants in genes that are key epigenetic regulators of spermatogenesis in the patients with non-obstructive azoospermia. Sci Rep.

[9] Jiao X, Ke H, Qin Y, Chen ZJ (2018). Molecular Genetics of Premature Ovarian Insufficiency. Trends Endocrinol Metab.

[10] Punab M, Poolamets O, Paju P, Vihljajev V, Pomm K, Ladva R (2017). Causes of male infertility: a 9-year prospective monocentre study on 1737 patients with reduced total sperm counts. Hum Reprod.

[11] Kasak L, Punab M, Nagirnaja L, Grigorova M, Minajeva A, Lopes AM (2018). Bi-allelic Recessive Loss-of-Function Variants in FANCM Cause Non-obstructive Azoospermia. Am J Hum Genet.

[12] Ayhan O, Balkan M, Guven A, Hazan R, Atar M, Tok A (2014). Truncating mutations in TAF4B and ZMYND15 causing recessive azoospermia. J Med Genet.

[13] Bestetti I, Barbieri C, Sironi A, Specchia V, Yatsenko SA, De Donno MD (2021). Targeted whole exome sequencing and Drosophila modelling to unveil the molecular basis of primary ovarian insufficiency. Hum Reprod.

[14] Veitia RA (2020). Primary ovarian insufficiency, meiosis and DNA repair. Biomed J.

[15] Guerri G, Maniscalchi T, Barati S, Busetto GM, Del Giudice F, De Berardinis E (2019). Non-syndromic monogenic male infertility. Acta Biomed.

[16] Gottlieb SF, Gulani A, Tegay DH (2021). Genetics, Meiosis. StatPearls. Treasure Island (FL).

[17] Gao J, Colaiacovo MP (2018). Zipping and Unzipping: Protein Modifications Regulating Synaptonemal Complex Dynamics. Trends Genet.

[18] Zhang FG, Zhang RR, Gao JM (2021). The organization, regulation, and biological functions of the synaptonemal complex. Asian J Androl.

[19] Hughes SE, Hawley RS (2020). Alternative Synaptonemal Complex Structures: Too Much of a Good Thing?. Trends Genet.

[20] Hollingsworth NM (2020). A new role for the synaptonemal complex in the regulation of meiotic recombination. Genes Dev.

[21] Wang S, Shang Y, Liu Y, Zhai B, Yang X, Zhang L (2021). Crossover patterns under meiotic chromosome program. Asian J Androl.

[22] von Diezmann L, Rog O (2021). Let's get physical - mechanisms of crossover interference. J Cell Sci.

[23] Pazhayam NM, Turcotte CA, Sekelsky J (2021). Meiotic Crossover Patterning. Front Cell Dev Biol.

[24] Syrjanen JL, Pellegrini L, Davies OR (2014). A molecular model for the role of SYCP3 in meiotic chromosome organisation. Elife.

[25] Bollschweiler D, Radu L, Joudeh L, Plitzko JM, Henderson RM, Mela I (2019). Molecular architecture of the SYCP3 fibre and its interaction with DNA. Open Biol.

[26] West AM, Rosenberg SC, Ur SN, Lehmer MK, Ye Q, Hagemann G (2019). A conserved filamentous assembly underlies the structure of the meiotic chromosome axis. Elife.

[27] Dunce JA-O, Dunne OM, Ratcliff M, Millán CA-O, Madgwick S, Usón I (2018). Structural basis of meiotic chromosome synapsis through SYCP1 self-assembly. Nat Struct Mol Biol.

[28] Dunce JM, Salmon LJ, Davies OR (2021). Structural basis of meiotic chromosome synaptic elongation through hierarchical fibrous assembly of SYCE2-TEX12. Nat Struct Mol Biol.

[29] Dunne OM, Davies OA-O (2019). Molecular structure of human synaptonemal complex protein SYCE1. Chromosoma.

[30] Sánchez-Sáez FA-O, Gómez HLA-O, Dunne OM, Gallego-Páramo C, Felipe-Medina NA-O, Sánchez-Martín MA-O (2020). Meiotic chromosome synapsis depends on multivalent SYCE1-SIX6OS1 interactions that are disrupted in cases of human infertility. Sci Adv.

[31] Gomez HL, Felipe-Medina N, Sanchez-Martin M, Davies OR, Ramos I, Garcia-Tunon I (2016). C14ORF39/SIX6OS1 is a constituent of the synaptonemal complex and is essential for mouse fertility. Nat Commun.

[32] Dunne OM, Davies OA-O (2019). A molecular model for self-assembly of the synaptonemal complex protein SYCE3. J Biol Chem.

[33] Lu J, Gu Y, Feng J, Zhou W, Yang X, Shen Y (2014). Structural insight into the central element assembly of the synaptonemal complex. Sci Rep.

[34] Pattabiraman D, Roelens B, Woglar A, Villeneuve AM (2017). Meiotic recombination modulates the structure and dynamics of the synaptonemal complex during C. elegans meiosis. PLoS Genet.

[35] Nadarajan S, Lambert TJ, Altendorfer E, Gao J, Blower MD, Waters JC (2017). Polo-like kinase-dependent phosphorylation of the synaptonemal complex protein SYP-4 regulates double-strand break formation through a negative feedback loop. Elife.

[36] Rog O, Kohler S, Dernburg AF (2017). The synaptonemal complex has liquid crystalline properties and spatially regulates meiotic recombination factors. Elife.

[37] Zickler D, Kleckner N (2015). Recombination, Pairing, and Synapsis of Homologs during Meiosis. Cold Spring Harb Perspect Biol.

[38] Takemoto K, Imai Y, Saito K, Kawasaki T, Carlton PM, Ishiguro KI (2020). Sycp2 is essential for synaptonemal complex assembly, early meiotic recombination and homologous pairing in zebrafish spermatocytes. PLoS Genet.

[39] Goodyer W, Kaitna S, Couteau F, Ward JD, Boulton SJ, Zetka M (2008). HTP-3 links DSB formation with homolog pairing and crossing over during C. elegans meiosis. Dev Cell.

[40] Cahoon CA-O, Helm JM, Libuda DA-O (2019). Synaptonemal Complex Central Region Proteins Promote Localization of Pro-crossover Factors to Recombination Events During Caenorhabditis elegans Meiosis. Genetics.

[41] Mu X, Murakami H, Mohibullah N, Keeney S (2020). Chromosome-autonomous feedback down-regulates meiotic DNA break competence upon synaptonemal complex formation. Genes Dev.

[42] Hillers KJ (2004). Crossover interference. Curr Biol.

[43] Ruiz-Herrera A, Vozdova M, Fernandez J, Sebestova H, Capilla L, Frohlich J (2017). Recombination correlates with synaptonemal complex length and chromatin loop size in bovids-insights into mammalian meiotic chromosomal organization. Chromosoma.

[44] Lynn A, Koehler KE, Judis L, Chan ER, Cherry JP, Schwartz S (2002). Covariation of synaptonemal complex length and mammalian meiotic exchange rates. Science.

[45] Frohlich J, Vozdova M, Kubickova S, Cernohorska H, Sebestova H, Rubes J (2015). Variation of Meiotic Recombination Rates and MLH1 Foci Distribution in Spermatocytes of Cattle, Sheep and Goats. Cytogenet Genome Res.

[46] Wang Y, Zhai B, Tan T, Yang X, Zhang J, Song M (2021). ESA1 regulates meiotic chromosome axis and crossover frequency via acetylating histone H4. Nucleic Acids Res.

[47] Song M, Zhai B, Yang X, Tan T, Wang Y, Yang X (2021). Interplay between Pds5 and Rec8 in regulating chromosome axis length and crossover frequency. Sci Adv.

[48] Lee MS, Higashide MT, Choi H, Li K, Hong S, Lee K (2021). The synaptonemal complex central region modulates crossover pathways and feedback control of meiotic double-strand break formation. Nucleic Acids Res.

[49] Libuda DE, Uzawa S, Meyer BJ, Villeneuve AM (2013). Meiotic chromosome structures constrain and respond to designation of crossover sites. Nature.

[50] Capilla-Perez L, Durand S, Hurel A, Lian Q, Chambon A, Taochy C (2021). The synaptonemal complex imposes crossover interference and heterochiasmy in Arabidopsis. Proc Natl Acad Sci U S A.

[51] France MG, Enderle J, Rohrig S, Puchta H, Franklin FCH, Higgins JD (2021). ZYP1 is required for obligate cross-over formation and cross-over interference in Arabidopsis. Proc Natl Acad Sci U S A.

[52] Cahoon CK, Hawley RS (2016). Regulating the construction and demolition of the synaptonemal complex. Nat Struct Mol Biol.

[53] Zhang Z, Xie S, Wang R, Guo S, Zhao Q, Nie H (2020). Multivalent weak interactions between assembly units drive synaptonemal complex formation. J Cell Biol.

[54] Schvarzstein M, Pattabiraman D, Bembenek JN, Villeneuve AM (2013). Meiotic HORMA domain proteins prevent untimely centriole disengagement during Caenorhabditis elegans spermatocyte meiosis. Proc Natl Acad Sci U S A.

[55] Kurdzo EL, Obeso D, Chuong H, Dawson DS (2017). Meiotic Centromere Coupling and Pairing Function by Two Separate Mechanisms in Saccharomyces cerevisiae. Genetics.

[56] Bisig CG, Guiraldelli MF, Kouznetsova A, Scherthan H, Hoog C, Dawson DS (2012). Synaptonemal complex components persist at centromeres and are required for homologous centromere pairing in mouse spermatocytes. PLoS Genet.

[57] Qiao H, Chen JK, Reynolds A, Hoog C, Paddy M, Hunter N (2012). Interplay between synaptonemal complex, homologous recombination, and centromeres during mammalian meiosis. PLoS Genet.

[58] Parra MT, Viera A, Gomez R, Page J, Benavente R, Santos JL (2004). Involvement of the cohesin Rad21 and SCP3 in monopolar attachment of sister kinetochores during mouse meiosis I. J Cell Sci.

[59] Previato de Almeida L, Evatt JM, Chuong HH, Kurdzo EL, Eyster CA, Gladstone MN (2019). Shugoshin protects centromere pairing and promotes segregation of nonexchange partner chromosomes in meiosis. Proc Natl Acad Sci U S A.

[60] Ichijima Y, Ichijima M, Lou Z, Nussenzweig A, Camerini-Otero RD, Chen J (2011). MDC1 directs chromosome-wide silencing of the sex chromosomes in male germ cells. Genes Dev.

[61] Turner JM (2007). Meiotic sex chromosome inactivation. Development.

[62] de la Fuente R, Pratto F, Hernandez-Hernandez A, Manterola M, Lopez-Jimenez P, Gomez R (2021). Epigenetic Dysregulation of Mammalian Male Meiosis Caused by Interference of Recombination and Synapsis. Cells.

[63] Abe H, Alavattam KG, Hu YC, Pang Q, Andreassen PR, Hegde RS (2020). The Initiation of Meiotic Sex Chromosome Inactivation Sequesters DNA Damage Signaling from Autosomes in Mouse Spermatogenesis. Curr Biol.

[64] Rappaport Y, Achache H, Falk R, Murik O, Ram O, Tzur YB (2021). Bisection of the X chromosome disrupts the initiation of chromosome silencing during meiosis in Caenorhabditis elegans. Nat Commun.

[65] Yuan L, Liu JG, Hoja MR, Wilbertz J, Nordqvist K, Hoog C (2002). Female germ cell aneuploidy and embryo death in mice lacking the meiosis-specific protein SCP3. Science.

[66] Yang F, De La Fuente R, Leu NA, Baumann C, McLaughlin KJ, Wang PJ (2006). Mouse SYCP2 is required for synaptonemal complex assembly and chromosomal synapsis during male meiosis. J Cell Biol.

[67] Xu H, Tong Z, Ye Q, Sun T, Hong Z, Zhang L (2019). Molecular organization of mammalian meiotic chromosome axis revealed by expansion STORM microscopy. Proc Natl Acad Sci U S A.

[68] Schilit SLP, Menon S, Friedrich C, Kammin T, Wilch E, Hanscom C (2020). SYCP2 Translocation-Mediated Dysregulation and Frameshift Variants Cause Human Male Infertility. American journal of human genetics.

[69] Lek M, Karczewski KJ, Minikel EV, Samocha KE, Banks E, Fennell T (2016). Analysis of protein-coding genetic variation in 60,706 humans. Nature.

[70] Stouffs K, Vandermaelen D, Tournaye H, Liebaers I, Lissens W (2011). Mutation analysis of three genes in patients with maturation arrest of spermatogenesis and couples with recurrent miscarriages. Reprod Biomed Online.

[71] Bolor H, Mori T, Nishiyama S, Ito Y, Hosoba E, Inagaki H (2009). Mutations of the SYCP3 gene in women with recurrent pregnancy loss. Am J Hum Genet.

[72] Miyamoto T, Hasuike S, Yogev L, Maduro MR, Ishikawa M, Westphal H (2003). Azoospermia in patients heterozygous for a mutation in SYCP3. Lancet.

[73] Fan S, Jiao Y, Khan R, Jiang X, Javed AR, Ali A (2021). Homozygous mutations in C14orf39/SIX6OS1 cause non-obstructive azoospermia and premature ovarian insufficiency in humans. American journal of human genetics.

[74] Gorsi B, Hernandez E, Moore MB, Moriwaki M, Chow CY, Coelho E (2021). Causal and Candidate Gene Variants in a Large Cohort of Women with Primary Ovarian Insufficiency. J Clin Endocrinol Metab.

[75] Hou D, Yao C, Xu B, Luo W, Ke H, Li Z (2021). Variations of C14ORF39 and SYCE1 identified in idiopathic premature ovarian insufficiency and nonobstructive azoospermia. J Clin Endocrinol Metab.

[76] Maor-Sagie E, Cinnamon Y Fau - Yaacov B, Yaacov B Fau - Shaag A, Shaag A Fau - Goldsmidt H, Goldsmidt H Fau - Zenvirt S, Zenvirt S Fau - Laufer N (2015). Deleterious mutation in SYCE1 is associated with non-obstructive azoospermia. J Assist Reprod Genet.

[77] Pashaei M, Rahimi Bidgoli MM, Zare-Abdollahi D, Najmabadi H, Haji-Seyed-Javadi R, Fatehi F (2020). The second mutation of SYCE1 gene associated with autosomal recessive nonobstructive azoospermia. J Assist Reprod Genet.

[78] de Vries L, Behar Dm Fau - Smirin-Yosef P, Smirin-Yosef P Fau - Lagovsky I, Lagovsky I Fau - Tzur S, Tzur S Fau - Basel-Vanagaite L, Basel-Vanagaite L (2014). Exome sequencing reveals SYCE1 mutation associated with autosomal recessive primary ovarian insufficiency. J Clin Endocrinol Metab.

[79] Hernández-López D, Geisinger A, Trovero MF, Santiñaque FF, Brauer M, Folle GA (2020). Familial primary ovarian insufficiency associated with an SYCE1 point mutation: defective meiosis elucidated in humanized mice. Mol Hum Reprod.

[80] McGuire MM, Bowden W, Engel NJ, Ahn HW, Kovanci E, Rajkovic A (2011). Genomic analysis using high-resolution single-nucleotide polymorphism arrays reveals novel microdeletions associated with premature ovarian failure. Fertil Steril.

[81] Jaillard S, Akloul L, Beaumont M, Hamdi-Roze H, Dubourg C, Odent S (2016). Array-CGH diagnosis in ovarian failure: identification of new molecular actors for ovarian physiology. J Ovarian Res.

[82] Bestetti I, Castronovo C, Sironi A, Caslini C, Sala C, Rossetti R (2019). High-resolution array-CGH analysis on 46,XX patients affected by early onset primary ovarian insufficiency discloses new genes involved in ovarian function. Hum Reprod.

[83] Tsuiko O, Noukas M, Zilina O, Hensen K, Tapanainen JS, Magi R (2016). Copy number variation analysis detects novel candidate genes involved in follicular growth and oocyte maturation in a cohort of premature ovarian failure cases. Hum Reprod.

[84] Franca MM, Funari MFA, Lerario AM, Santos MG, Nishi MY, Domenice S (2020). Screening of targeted panel genes in Brazilian patients with primary ovarian insufficiency. PLoS One.

[85] Zhe J, Ye D, Chen X, Liu Y, Zhou X, Li Y (2020). Consanguineous Chinese Familial Study Reveals that a Gross Deletion that Includes the SYCE1 Gene Region Is Associated with Premature Ovarian Insufficiency. Reprod Sci.

[86] An M, Liu Y, Zhang M, Hu K, Jin Y, Xu S (2021). Targeted next-generation sequencing panel screening of 668 Chinese patients with non-obstructive azoospermia. J Assist Reprod Genet.

[87] Geisinger A, Benavente R (2016). Mutations in Genes Coding for Synaptonemal Complex Proteins and Their Impact on Human Fertility. Cytogenet Genome Res.

[88] Bolcun-Filas E, Hall E, Speed R, Taggart M, Grey C, de Massy B (2009). Mutation of the mouse Syce1 gene disrupts synapsis and suggests a link between synaptonemal complex structural components and DNA repair. PLoS Genet.

[89] Karczewski KJ, Francioli LC, Tiao G, Cummings BB, Alfoldi J, Wang Q (2020). The mutational constraint spectrum quantified from variation in 141,456 humans. Nature.

[90] Liu Y, Zhao Q, Nie H, Zhang F, Fu T, Zhang Z (2021). SYP-5 regulates meiotic thermotolerance in Caenorhabditis elegans. J Mol Cell Biol.

[91] Luck K, Sheynkman GM, Zhang I, Vidal M (2017). Proteome-Scale Human Interactomics. Trends Biochem Sci.

[92] Vidal M, Cusick ME, Barabasi AL (2011). Interactome networks and human disease. Cell.

[93] Luck K, Kim DK, Lambourne L, Spirohn K, Begg BE, Bian W (2020). A reference map of the human binary protein interactome. Nature.

[94] Zhang QH, Qi ST, Wang ZB, Yang CR, Wei YC, Chen L (2012). Localization and function of the Ska complex during mouse oocyte meiotic maturation. Cell Cycle.

[95] Golubkova EV, Markova EG, Markov AV, Avanesyan EO, Nokkala S, Mamon LA (2009). Dm nxf1/sbr gene affects the formation of meiotic spindle in female Drosophila melanogaster. Chromosome Res.

[96] Lange BM, Rebollo E, Herold A, Gonzalez C (2002). Cdc37 is essential for chromosome segregation and cytokinesis in higher eukaryotes. EMBO J.

[97] Huang M, Zhu M, Jiang T, Wang Y, Wang C, Jin G (2019). Fine mapping the MHC region identified rs4997052 as a new variant associated with nonobstructive azoospermia in Han Chinese males. Fertil Steril.

[98] Zhang Z, Li W, Zhang Y, Zhang L, Teves ME, Liu H (2016). Intraflagellar transport protein IFT20 is essential for male fertility and spermiogenesis in mice. Mol Biol Cell.

[99] Kim J, Kwon JT, Jeong J, Kim J, Hong SH, Kim J (2018). SPATC1L maintains the integrity of the sperm head-tail junction. EMBO Rep.

[100] Mendoza-Lujambio I, Burfeind P, Dixkens C, Meinhardt A, Hoyer-Fender S, Engel W (2002). The Hook1 gene is non-functional in the abnormal spermatozoon head shape (azh) mutant mouse. Hum Mol Genet.

[101] Gao A, Shrinivas K, Lepeudry P, Suzuki HI, Sharp PA, Chakraborty AK (2018). Evolution of weak cooperative interactions for biological specificity. Proc Natl Acad Sci U S A.

[102] Yuan L, Liu JG, Zhao J, Brundell E, Daneholt B, Hoog C (2000). The murine SCP3 gene is required for synaptonemal complex assembly, chromosome synapsis, and male fertility. Mol Cell.

[103] de Vries FA, de Boer E, van den Bosch M, Baarends WM, Ooms M, Yuan L (2005). Mouse Sycp1 functions in synaptonemal complex assembly, meiotic recombination, and XY body formation. Genes Dev.

[104] Bolcun-Filas E, Costa Y, Speed R, Taggart M, Benavente R, De Rooij DG (2007). SYCE2 is required for synaptonemal complex assembly, double strand break repair, and homologous recombination. J Cell Biol.

[105] Schramm S, Fraune J, Naumann R, Hernandez-Hernandez A, Hoog C, Cooke HJ (2011). A novel mouse synaptonemal complex protein is essential for loading of central element proteins, recombination, and fertility. PLoS Genet.

[106] Hamer G, Wang H, Bolcun-Filas E, Cooke HJ, Benavente R, Hoog C (2008). Progression of meiotic recombination requires structural maturation of the central element of the synaptonemal complex. J Cell Sci.

[107] Mizutani E, Suzumori N, Ozaki Y, Oseto K, Yamada-Namikawa C, Nakanishi M (2011). SYCP3 mutation may not be associated with recurrent miscarriage caused by aneuploidy. Hum Reprod.

[108] Sazegari A, Kalantar SM, Pashaiefar H, Mohtaram S, Honarvar N, Feizollahi Z (2014). The T657C polymorphism on the SYCP3 gene is associated with recurrent pregnancy loss. J Assist Reprod Genet.

